# Role of tumor size in the pre-operative management of rectal cancer patients

**DOI:** 10.1186/1471-230X-10-61

**Published:** 2010-06-15

**Authors:** Inti Zlobec, Parham Minoo, Eva Karamitopoulou, George Peros, Efstratios S Patsouris, Frank Lehmann, Alessandro Lugli

**Affiliations:** 1 Institute of Pathology, University Hospital of Basel, Basel, Switzerland; 2 Department of Pathology, University of California San Diego, San Diego, CA, USA; 3 Second Department of Pathology, University of Athens, Athens, Greece; 4 Fourth Department of Surgery, University of Athens, Athens, Greece; 5 First Department of Pathology, University of Athens, Athens, Greece; 6 Department of Gastroenterology, University Hospital of Basel, Basel, Switzerland

## Abstract

**Background:**

Clinical management of rectal cancer patients relies on pre-operative staging. Studies however continue to report moderate degrees of over/understaging as well as inter-observer variability. The aim of this study was to determine the sensitivity, specificity and accuracy of tumor size for predicting T and N stages in pre-operatively untreated rectal cancers.

**Methods:**

We examined a test cohort of 418 well-documented patients with pre-operatively untreated rectal cancer admitted to the University Hospital of Basel between 1987 and 1996. Classification and regression tree (CART) and logistic regression analysis were carried out to determine the ability of tumor size to discriminate between early (pT1-2) and late (pT3-4) T stages and between node-negative (pN0) and node-positive (pN1-2) patients. Results were validated by an external patient cohort (n = 28).

**Results:**

A tumor diameter threshold of 34 mm was identified from the test cohort resulting in a sensitivity and specificity for late T stage of 76.3%, and 67.4%, respectively and an odds ratio (OR) of 6.67 (95%CI:3.4-12.9). At a threshold value of 29 mm, sensitivity and specificity for node-positive disease were 94% and 15.5%, respectively with an OR of 3.02 (95%CI:1.5-6.1). Applying these threshold values to the validation cohort, sensitivity and specificity for T stage were 73.7% and 77.8% and for N stage 50% and 75%, respectively.

**Conclusions:**

Tumor size at a threshold value of 34 mm is a reproducible predictive factor for late T stage in rectal cancers. Tumor size may help to complement clinical staging and further optimize the pre-operative management of patients with rectal cancer.

## Background

Clinical management of patients with rectal cancer depends significantly on pre-operative staging. Parameters such as cT and cN stage obtained by magnetic resonance imaging (MRI), computed tomography (CT) or endorectal ultrasonography (EUS) are crucial in selecting patients for pre-operative neoadjuvant therapy [1,2]. According to the European Society of Medical Oncology (ESMO), patients with T1-2, some early T3 and lymph node-negative disease may benefit most from surgery alone, whereas patients with more locally advanced stages (most T3, some T4 and those with lymph node positivity) are recommended for pre-operative radiotherapy followed by total mesorectal excision (TME) with the goal of decreasing local recurrence rates [3,4].

However, recent studies report variability in the accuracy of pre-operative staging of rectal cancer. All three methods (CT, MRI and EUS) can lead to moderate rates of over- or understaging of T and N stages compared to histology in pre-operatively untreated patients [5-10]. Whereas EUS or CT seem to be more accurate for the detection of early T1/T2 cancer, understaging with CT has been described for T3 tumors in comparison to MRI [10-13]. In contrary, lymph node positivity may be best detected by MRI, although inter-observer variability using this method has been described [14-19]. Overall, clinical imaging appears to result in an overall accuracy of 65%-90% and is closely related to observer experience [1]. Taken together, detection of novel prognostic factors capable of complementing clinical staging is warranted to identify patients in the pre-operative setting with locally advanced disease.

We and other groups have suggested that protein biomarkers in the pre-operative biopsy may help to identify patients with poor survival who might be candidates for neoadjuvant therapy [20]. Such biomarkers include the epidermal growth factor receptor (EGFR) and vascular endothelial growth factor (VEGF), among others [21-23]. To date, despite promising results, no single immunohistochemical protein marker has been introduced into daily practice. This may in large part be due to the lack of standardized scoring systems for evaluating immunohistochemistry results.

The tumor diameter plays an integral part in cancer staging in certain tumor types, such as stage I to III breast cancer and gastric cancer [24]. In the latter group, tumor size has been reported to be an independent prognostic factor of both pT and pN stages [25-29]. In fact, the strong link between tumor size, pT and pN stages has also been used to provide evidence supporting the cause of death of Emperor Napoleon Bonaparte from gastric cancer [30].

Only limited data have been published regarding the role of tumor size in predicting T and N stages in untreated rectal cancer patients. If an association could be demonstrated, it would provide crucial information for clinical staging. The aim of this study was to determine the sensitivity, specificity and accuracy of tumor size for predicting T and N stages in rectal cancers. To this end, we evaluated 418 patients with rectal cancers who received no pre-operative therapy, therefore tumor size and its predictive value was elaborated based on pT and pN stages confirmed pathologically. Results were subsequently validated by an external patient cohort.

## Methods

### Test Cohort

482 untreated, unselected rectal cancer patients admitted to the University Hospital of Basel between 1987 and 1996 were initially included in this study. Haematoxylin and eosin (H&E) stained slides were retrospectively collected from the Institute of Pathology, University Hospital of Basel, the Institute of Clinical Pathology, Basel, Switzerland and the Institute of Pathology, Stadtspital Triemli, Zürich, Switzerland. Histopathological criteria were reviewed by an experienced gastrointestinal pathologist and included tumor diameter, pT and pN classification, grade of differentiation, histologic subtype, and the presence of tumor invasion into vessels. Clinical data including patient age at diagnosis, tumor location and follow-up were obtained from patient records. Cancer-specific survival time was the main clinical endpoint of interest. Censored observations included patients who were alive at the last follow-up, died for reasons other than colorectal cancer or were lost to follow-up. 5-year cancer-specific survival rate was 57% (95%CI: 51-61). Patient characteristics are summarized in Table 1. The use of patient data has been approved by the local Ethics Committee of the University of Basel, Switzerland.

**Table 1:** Characteristics of rectal cancer patients in the test cohort (n = 418)

Clinco-pathological features		Frequency N (%)
Patient age at diagnosis (years)	Median (min-max)	69.0 (36-96)
		
Tumor size (mm)	Median (min-max)	45.0 (12-100)
		
Gender	Male	197 (47.1)
	Female	221 (52.9)
		
Diagnosis	Non-mucinous	18 (4.3)
	Mucinous	400 (95.7)
		
pT classification	pT1-2	119 (28.5)
	pT3-4	299 (71.5)
		
pN classification	pN0	226 (54.1)
	pN1-2	192 (45.9)
		
Tumor grade	G1-2	392 (94.2)
	G3	24 (5.8)
		
Vascular invasion	Absent	311 (74.8)
	Present	105 (25.2)
		
Distant metastasis	Absent	77 (90.6)
	Present	8 (9.4)
		
Post-operative therapy	None	66 (77.7)
	Treated	19 (22.4)
		
Survival rate (5-years)	95%CI	56.9 (52-62)

### External Validation Cohort

28 non-consecutive rectal cancer patients treated at the 4th Department of Surgery, University of Athens Medical School were randomly selected from the archives of the 2nd Department of Pathology, University of Athens Medical School (Attikon University Hospital), Greece. Patients were treated between 2004 and 2006. All histomorphological data were reviewed from the corresponding hematoxylin and eosin (H&E) stained slides, while clinical data were obtained from corresponding reports. Information included gender, age, tumor diameter, histological subtype, tumor location, pT stage, pN stage, pM stage, vascular invasion and lymphatic invasion. 5-year survival rate for the entire cohort was 67.6% (44-83). Information on post-operative therapy was available for all patients. Patient characteristics are summarized in Table 2. The use of patient data has been approved by the local Ethics Committee of the University of Athens, Greece.

**Table 2:** Characteristics of rectal cancer patients in the validation cohort (n = 28)

Clinco-pathological features		Frequency N (%)
Patient age at diagnosis (years)	Median (min-max)	65 (38-82)
		
Tumor size (mm)	Median (min-max)	40 (10-70)
		
Gender	Male	13 (46.4)
	Female	15 (53.6)
		
Diagnosis	Non-mucinous	26 (92.9)
	Mucinous	2 (7.1)
		
pT classification	pT1-2	12 (42.9)
	pT3-4	16 (57.1)
		
pN classification	pN0	15 (53.6)
	pN1-2	13 (46.4)
		
pM classification	pM0	27 (96.4)
	pM1	1 (3.6)
		
Tumor grade	G1-2	18 (78.3)
	G3	5 (21.7)
		
Vascular or lymphatic invasion	Absent	25 (89.3)
	Present	3 (10.7)
		
Adjuvant therapy	Absent	10 (35.7)
	Present	18 (64.3)
		
Survival rate (5-years)	95%CI	67.6 (44-83)

### Statistical Analysis

To determine the most appropriate cut-off score for tumor diameter and to allow optimal classification of patients into pT1-2 and pT3-pT4 as well as pN0 and pN1-2 stages, Classification and Regression Tree (CART) analysis was performed on the test group of 418 patients. CART generates a clinical decision rule which can be visualized as a "decision-tree". The computer-generated algorithm uses a binary recursive process which splits the data into the best possible combination of variables to optimally classify patients into those with or without the outcome. To measure the classification error as a function of tree size, 10-fold cross-validation experiments were performed. For each of those experiments, data were randomly split into 10 smaller subsets. A backward pruning method was used to choose the best number of nodes from the original tree. Once the 10 trees are built, their classification error rate as a function of tree size is averaged. The tree size that produces the least amount of misclassification is selected as the optimal tree. Simple logistic regression analysis was performed in order to determine the odds of having pT3-4 or pN1-2 with tumors below or above the obtained cut-off score from CART analysis. Odds ratios (OR) and 95% confidence intervals (CI) were used to determine the effect of tumor diameter on more advanced pT or pN classification. Finally, receiver operating characteristic (ROC) curve analysis and the area under the ROC curve (AUC) were used to determine overall accuracy of tumor size on outcome. The closer the AUC value is to 1.0 and the further away from 0.5, the more tumor size discriminates between pT and pN classification. Kaplan-Meier survival curve and the log-rank test were used to evaluate survival time differences in univariate setting. P-values <0.05 were considered statistically significant.

## Results

### Test Cohort

#### T stage

**Figure 1:**
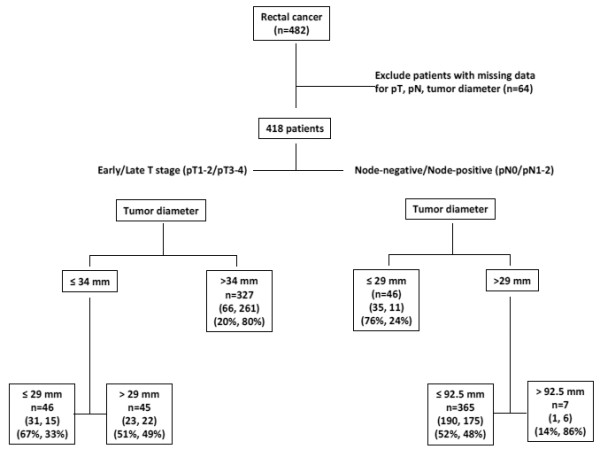
**Classification of rectal cancer patients (test cohort) into early and late T stages or into node-negative and node-positive disease based on tumor diameter using classification and regression tree analysis (CART)**. For the classification of patients by T stage, numbers in parentheses describe (the number of patients with early T, late T stage) followed by (percentage of patients with early T and late T stages). For the classification of patients by N stage, numbers in parentheses describe (the number of patients with node-negative, node-positive disease) followed by (percentage of patients with node-negative, node-positive disease).

Of the 482 patients initially included in our study, information on tumor diameter, pT and pN stages was available in 418 cases. Using CART analysis, a tumor diameter of 34 mm was found to be most useful for discriminating between patients with early (pT1/pT2) and late (pT3/pT4) T stage. In particular, of the 327 cases with >34 mm rectal cancer, 80% (n = 261) had late T stage cancers. These patients had a 6.67 (95%CI: 3.4-12.9) times greater odds of pT3-4 lesions compared to those with ≤34 mm (Table 3). Moreover, 31/46 (67%) patients with ≤29 mm tumors in diameter were pT1-pT2 (Figure 1). 45 patients had tumors with a diameter of 29-34 mm. Classification of these patients into early or late T stages was ambiguous with 51% found to be early and 49% late T stage. Sensitivity and specificity of tumor size for pT3-4 using a cut-off score of 34 mm were 76.3% and 67.4%, respectively, with an overall diagnostic accuracy of 66%.

**Table 3:** Association of tumor diameter with pT and pN classifications in rectal cancer-test cohort (n = 418)

		Frequency N (%)		OR (95%CI)	P-value	AUC
						
		≤ 34 mm	>34 mm			
						
pT classification	pT1-2	31 (67.4)	88 (23.7)	6.67 (3.4-12.9)	<0.001	0.66
	pT3-4	15 (32.6)	284 (76.3)			
						
		≤ 29 mm	>29 mm			
						
pN classification	pN0	35 (15.5)	11 (5.7)	3.02 (1.5-6.1)	0.002	0.55
	pN1-2	191 (84.5)	181 (94.3)			

OR: odds ratio. AUC: area under the ROC curve, illustrating discriminatory ability of tumor size for pT and pN classifications

### N stage

A tumor diameter of 29 mm was most discriminating between node-negative (pN0) and node-positive (pN1-2) patients. Of the 46 patients with ≤29 mm cancers, 76% (n = 35) had node-negative and 24% node-positive disease. These patients had an OR of 3.02 (95%CI: 1.5-6.1) indicating that cases >29 mm in diameter had more than a 3-fold greater odds of node-positivity compared to cancers that were ≤29 mm. Only 7 patients had tumors exceeding 92.5 mm in size and 6/7 (85.7%) were node-positive. A large subgroup of patients (n = 365) with tumor diameters from 29 to 92.5 mm had a similar probability of being classified as either node-negative (52%; n = 190) or node-positive (48%, n = 175). Although highly sensitive for pN1-2 stages, tumor size was significantly less specific for pN0 cases (sensitivity and specificity for pN1-2 were 94.3% and 15.5%, respectively), while the overall diagnostic accuracy was 55%.

#### Comparison of survival by T stage and tumor size

**Figure 2:**
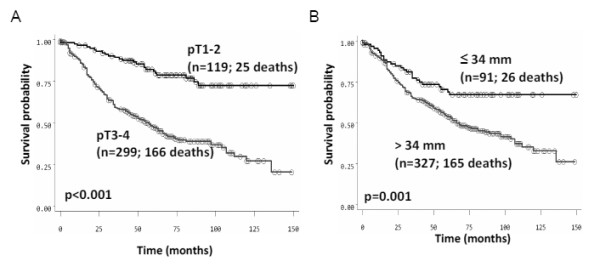
**Kaplan-Meier survival curves showing differences in survival time between patients with (A) early (pT1-2) and late (pT3-4) rectal tumors and with (B) ≤34 mm and >34 mm rectal tumors**.

The predictive ability of a tumor diameter of 34 mm for early and late T stages was validated using survival time. In Figure 2, survival time differences between patients with early and late pT stages are shown (p < 0.001). Similar differences were observed when compared to survival time differences in patients with ≤34 or >34 mm tumor sizes (p = 0.001).

#### External Validation Cohort

To validate these findings, a second independent cohort of untreated rectal cancer patients was analyzed. Applying a cut-off of 34 mm to tumor size and classifying patients in early or late T stages, the sensitivity, specificity, and overall diagnostic accuracy for late T stage were 73.7%, 77.8% and 73%, respectively (Table 4). For pN classification, using a cut-off of 29 mm, sensitivity, specificity and overall accuracy were 50%, 75% and 56%, respectively. Thus, the high accuracy of tumor size in predicting T stages and its moderate ability with respect to N stages were reproduced in this second cohort.

**Table 4:** Association of tumor diameter with pT and pN classifications in rectal cancer- validation cohort (n = 28)

		Frequency N (%)		OR (95%CI)	P-value	AUC
		≤ 34 mm	>34 mm			
pT classification	pT1-2	7 (77.8)	5 (26.3)	9.8 (1.5-64)	0.017	0.73
	pT3-4	2 (22.2)	14 (73.7)			
		≤ 29 mm	>29 mm			
pN classification	pN0	3 (75.0)	12 (50.0)	3.0 (0.3-33.1)	0.37	0.56
	pN1-2	1 (25.0)	12 (50.0)			

OR: odds ratio. AUC: area under the ROC curve, illustrating discriminatory ability of tumor size for pT and pN classifications

## Discussion and conclusions

The results of this study using test and validation cohorts of more than 400 pre-operatively untreated rectal cancer patients suggest that tumor size at a cut-off of 34 mm is significantly related to pT stage. This indicates that tumor size can be used to improve the assessment of pre-operative staging.

Precise pre-operative staging has a crucial role in planning treatment strategy in rectal cancer. Extensive studies have compared the accuracy of different imaging modalities to correctly stage rectal cancers pre-operatively. These studies show that despite considerable improvement in resolution of various imaging techniques, accurate pre-operative staging remains challenging. For example, differentiation between T2 and early T3 stages is often difficult even with MRI due to desmoplastic reaction around the tumor that mimics neoplastic tissue [31-34]. Inability to identify lymph nodes containing micrometastases as well as size overlap between reactive nodes and those containing metastases are other major challenges for all imaging modalities including high resolution MRI [33,35-37].

Previous reports have evaluated the association of tumor size, prognosis, cancer recurrence and peri-rectal lymph node involvement. Wolmark and colleagues found that among patients with Dukes C colorectal tumors, depth of penetration was related to tumor size and number of positive lymph nodes [38]. In a comprehensive analysis, Cai and colleagues, reported an independent predictive effect of tumor size along with pre-operative CEA level and tumor differentiation for identifying locally advanced rectal cancer [39]. In a series of 265 pT1 and pT2 colorectal cancer patients including 164 rectal cancers, Chock and Law showed that tumor size was associated with pT stage but not lymph node status [40]. A similar study by Rasheed et al on 303 patients with pT1 and pT2 rectal cancer revealed a direct correlation between tumor size and depth of invasion but not lymph node metastasis [41]. Kikuchi et al reported a direct association between tumor diameter and depth of invasion as well as lack of a correlation between tumor size and lymph node metastasis in 182 colorectal cancer cases [42]. Using 10 mm as cut off, Matsuda et al showed that depth of invasion but not node involvement was associated with tumor size [43]. Our study suggests for the first time a tumor diameter threshold allowing reproducible prediction of higher T stages (pT3-pT4) and worse survival. The results of this study are in line with most previous reports showing lack of accuracy for tumor size to predict N stage [44-47]. The study of Zhang et al is among the few reports demonstrating a direct association between tumor size and nodal metastasis [48].

Our study is limited by the fact that it is a retrospective analysis of rectal cancer patients. Additionally, since TME was only introduced in Switzerland for the treatment of rectal cancer in the summer of 1995, nearly all the patients in this study did not undergo this surgical procedure. Thirdly, the assessment of tumor deposits, as required for accurate staging according to the most recent 7th edition of the American Joint Committee on Cancer and International Union against Cancer Classification (AJCC/UICC) were not evaluated in this series; staging was performed according to the 6th edition of the cancer staging manual. However, since this study was not focused on prognosis of patients with rectal cancer but rather on the predictive ability of tumor size on pT and pN stage, the lack of information of these and other prognostic factors only minimally affects the major results of this study. In addition, this analysis benefits from a large number of rectal cancer patients as well as the use of a test and validation group. The results of the latter confirmed that the determined cut-off score of 34 mm leads to similar prediction of advanced pT stage.

In conclusion, tumor size may help to determine the best treatment option for rectal cancer patients. A tumor size of >34 mm is highly related to more advanced pT stages.

## Competing interests

The authors declare that they have no competing interests.

## Authors' contributions

IZ, PM and AL contributed to conception and design, manuscript drafting, analysis and data interpretation; EK and FL made critical revisions for intellectual content and data interpretation while EP and GP contributed to data acquisition. All authors gave their approval of the final manuscript.

## Pre-publication history

The pre-publication history for this paper can be accessed here:

http://www.biomedcentral.com/1471-230X/10/61/prepub
